# Acidic environment causes apoptosis by increasing caspase activity

**DOI:** 10.1038/sj.bjc.6690617

**Published:** 1999-08

**Authors:** H J Park, J C Lyons, T Ohtsubo, C W Song

**Affiliations:** Department of Therapeutic Radiology–Radiation Oncology, University of Minnesota Medical School, 420 Delaware St SE, Box 494 Mayo, Minneapolis, MN 55455, USA; College of Medicine, Department of Microbiology, Inha University, 253 Yong-Hyun Dong, Nam-Gu, Inchon, 402-751, Korea

**Keywords:** apoptosis, acidic stress, pH_i_, caspases, PARP cleavage, Bax

## Abstract

An exposure of HL-60 human promyelocytic leukaemia cells to acidic media with pH 6.2–6.6 caused an up-regulation of Bax protein expression within 2 h, which lasted for longer than 6 h. On the other hand, the apoptosis, as judged from PARP cleavage, DNA fragmentation and flow cytometric determination of cell population with sub-G1 DNA content, occurred after the cells were incubated in the acidic media for longer than 4 h. The PARP cleavage and DNA fragmentation in the cells exposed to an acidic environment could be effectively suppressed by inhibitors specific for ICE or CPP32, indicating that activation of these caspases is an essential step in acidic stress-induced apoptosis. It has been known that Bax is involved in the activation of caspases. Taken together, it appears that acidic stress first up-regulates Bax protein thereby activating caspases followed by PARP cleavage and DNA fragmentation. The observation that inhibition of either ICE or CPP32 could suppress acidic stress-induced apoptosis suggested that ICE activates pro-CPP32, which then cleaves PARP. Flow cytometric analysis indicated that acidic stress-induced apoptosis occurs mainly in G1 cells. The finding in the present study demonstrated that acidic intra-tumour environment may markedly perturb the tumour cell proliferation and tumour growth. © 1999 Cancer Research Campaign


*British Journal of Cancer* (1999) **80**(12), 1892–1897
© 1999 Cancer Research Campaign
Article no. bjoc.1999.0617

### Acidic environment causes apoptosis by increasing caspase activity

**HJ Park[1,2], JC Lyons[1], T Ohtsubo[1] and CW Song[1]**

[1]University of Minnesota Medical School, Department of Therapeutic Radiology–Radiation Oncology, 420 Delaware St SE, Box 494 Mayo, Minneapolis, MN 55455, USA; [2]Inha University, College of Medicine, Department of Microbiology, 253 Yong-Hyun Dong, Nam-Gu, Inchon 402-751, Korea

**Summary** An exposure of HL-60 human promyelocytic leukaemia cells to acidic media with pH 6.2–6.6 caused an up-regulation of Bax protein expression within 2 h, which lasted for longer than 6 h. On the other hand, the apoptosis, as judged from PARP cleavage, DNA fragmentation and flow cytometric determination of cell population with sub-G1 DNA content, occurred after the cells were incubated in the acidic media for longer than 4 h. The PARP cleavage and DNA fragmentation in the cells exposed to an acidic environment could be effectively suppressed by inhibitors specific for ICE or CPP32, indicating that activation of these caspases is an essential step in acidic stress-induced apoptosis. It has been known that Bax is involved in the activation of caspases. Taken together, it appears that acidic stress first up-regulates Bax protein thereby activating caspases followed by PARP cleavage and DNA fragmentation. The observation that inhibition of either ICE or CPP32 could suppress acidic stress-induced apoptosis suggested that ICE activates pro-CPP32, which then cleaves PARP. Flow cytometric analysis indicated that acidic stress-induced apoptosis occurs mainly in G1 cells. The finding in the present study demonstrated that acidic intra-tumour environment may markedly perturb the tumour cell proliferation and tumour growth.

**Keywords**: apoptosis; acidic stress; $pH_i$; caspases; PARP cleavage; Bax

It has been known that the intra-tumour environment is acidic with pH as low as 5.6 due to high glycolytic activity of malignant cells, insufficient vascular supply and sluggish blood circulation (Aisenberg, 1961; Gullino, 1975; Wike-Hooley et al, 1984; Griffiths, 1991; Song et al, 1993*a*). Since various metabolic pathways are directly influenced by acidity, an acidic intra-tumour environment would significantly affect viability and proliferation of tumour cells. Indeed, it has been demonstrated that intracellular acidification by exposing cells to an acidic environment or by interfering with the intracellular pH ($pH_i$) control mechanisms causes cellular damage and sensitizes cells to chemotherapy or hyperthermia (Haveman, 1979; Chu and Dewey, 1988; Jahde et al, 1989; Kim et al, 1991; Song et al, 1993*a*, 1993*b*, 1994; Liu and Fox, 1995; Liu et al, 1996; Lee et al, 1997; Takasu et al, 1998). Tannock and Rotin (1989) reviewed the influence of pH on cell viability and on the activity of therapeutic agents and demonstrated that the $pH_i$ regulatory mechanism may be used as a target of therapy exploiting the fact that the interstitial environment in tumours is markedly acidic as compared with that in normal tissues. We have observed that an exposure of cells to acidic medium induces apoptosis and that an acidification of the intracellular environment by interfering with the intracellular pH ($pH_i$) regulatory mechanisms induces apoptosis even in a neutral extracellular pH ($pH_e$) environment (Park et al, 1996; Lee et al, 1997).

Proteolytic degradation of various vital proteins including PARP (poly (ADP-ribose) polymerase) by ICE (interleukin-1β converting enzyme)-like caspases is a common effector phase in apoptosis caused by various signals. Degradation of each protein appears to be carried out by one or more caspases specific for each protein (Lazebnick et al, 1994; Nicholson et al, 1995; Whitacre et al, 1995; Kumar and Lavin, 1996; Schlegel et al, 1996). For example, PARP is cleaved mainly by CPP32. Furlong et al (1997) recently reported that $pH_i$ was decreased, PARP was cleaved and DNA was fragmented during apoptosis in IL-3-dependent BAF3 cells after withdrawal of IL-3 or treatment with topoisomerase inhibitor etoposide. These investigators concluded that the intracellular acidification triggered apoptosis by directly or indirectly activating ICE-like proteases. On the other hand, Wolf et al (1997) reported that acidification during apoptosis is downstream of ICE-like protease activation, suggesting that the PARP cleavage by caspases during apoptosis is not caused by intracellular acidification. In the present report we describe our observation that in HL-60 human promyelocytic leukaemia cells, acidic stress causes apoptosis first by up-regulating a pro-apoptotic factor, Bax, thereby activating ICE-like caspases.

*Received 23 November 1998*
*Revised 4 March 1999*
*Accepted 9 March 1999*

*Correspondence to:* HJ Park

### MATERIALS AND METHODS

### Cell line and culture conditions

Exponentially growing HL-60 human promyelocytic leukaemia cells were used in this study. The cells were cultured under a humidified 5% carbon dioxide/95% air atmosphere at 37°C. The cell density was maintained at fewer than $3 \times 10^5$ cells $ml^{-1}$ in 25-$cm^2$ plastic tissue culture flasks with 10 ml of RPMI-1640 culture medium supplemented with 10% (v/v) fetal calf serum. Apoptosis was induced by incubating the cells in fresh medium that had been adjusted to the desired pHs using 30 mM each of Tris, MOPS

(3-(4-morpholino)propanesulphonic acid) and MES (morpholinoethanesulphonic acid) buffers.

### DNA gel electrophoresis

The cells were collected by centrifugation, washed with phosphate-buffered saline (PBS) and resuspended in lysis buffer (10 mM Tris–HCl, pH 7.4; 10 mM sodium chloride (NaCl); 10 mM EDTA; proteinase K at 0.1 mg $ml^{-1}$; 0.5% (w/v) sodium dodecyl sulphate (SDS)) and incubated at 48°C overnight. Cold (4°C) 5 M NaCl solution was added to the lysate and this mixture was vigorously shaken with a vortex and centrifuged at 10 000 ***g*** for 5 min. The supernatant was mixed with isopropanol (1:1) and incubated at –20°C overnight. The pellet was resuspended in TE buffer (10 mM Tris–HCl pH 7.4, 1 mM EDTA) and the RNA was digested by adding 0.2 mg $ml^{-1}$ DNAase-free RNAase. Twenty micrograms of DNA from each sample were subjected to electrophoresis on 1.5% agarose gel in TBE (89 mM Tris base, 89 mM boric acid, 2 mM EDTA) and the DNA was stained with ethidium bromide (Park et al, 1996; Lee et al, 1997; Takasu et al, 1998).

### Western blotting analysis of PARP, Bcl-2 and Bax

Cells were collected by centrifugation, washed with PBS and dissolved in solubilizing buffer (pH 7.4, 1% Triton X-100, 1% deoxycholic acid sodium salt, 0.1% SDS, 20 mM Tris–HCl, 150 mM NaCl, 5 mM EDTA, 1 mM phenylmethylsulphonyl fluoride, 10 μg $ml^{-1}$ aprotinin and 10 μg $ml^{-1}$ leupeptin). Twenty-five micrograms of protein per lane were electrophoresed on 7.5% polyacrylamide gels. The proteins were visualized by staining with 0.1% Coomassie brilliant blue R-250 in a solution of 50% methanol and 10% acetic acid in water. The gels were destained in the same methanol–acetic acid solution without bromophenol blue and then transblotted to Hybond-P (Amersham, Arlington Heights, IL, USA) in transfer buffer (192 mM glycine, 25 mM Tris, 2.5 mM SDS, 10% methanol). The blots were blocked with 3% non-fat, dry milk in TBST (pH 7.4), incubated with anti-PARP antibody (1:1000 dilution; UBI), Bcl-2 (1:1000 dilution; UBI) and Bax (0.5 μg $ml^{-1}$; UBI), respectively, and then treated with a horseradish peroxidase (HRP)-conjugated anti-rabbit IgG antibody. The immunoreactive bands were visualized using chemiluminescence (Takasu et al, 1998).

### Effect of caspase inhibitors on apoptosis

The ICE inhibitor, Ac-YVAD-H (Peptide International, Louisville, KY, USA) and CPP32 inhibitor, Ac-DEVD-H (Peptide International) were dissolved in dimethyl sulphoxide and added to HL-60 cells in pH 7.5 medium to a final concentration of 100 μM. After incubating the cells with the inhibitors at 37°C for 1 h, the inhibitors were removed by washing the cells with pH 7.5 medium. The drug-treated cells were resuspended in various pH media and incubated for 4 h at 37°C. PARP cleavage and DNA fragmentation were then determined.

### Flow cytometric analysis

Occurrence of apoptosis and changes in cell cycle distribution were analysed with the flow cytometry method (Darzynkiewicz et al, 1992; Lee et al, 1997). Cells were fixed in 10 ml cold 80% (v/v) ethanol at 4°C overnight. The cells were then centrifuged, washed with 1 ml PBS and resuspended in 2 ml PBS. To a 2 ml cell suspension, 30 units of DNAase-free RNAase was added and then 100 μl PI (propidium iodide; 50 μg $ml^{-1}$) were added. After a gentle mixing, the resuspended cells were incubated under a dark condition at 37°C for 1 h and covered until used. The PI fluorescence of the cells was measured using about $2 \times 10^4$ cells in a FACSCalibur flow cytometer (Becton Dickinson, San Jose, CA, USA). The fraction of cells in various cell cycle stages and apoptosis was estimated from the cellular DNA content (Takasu et al, 1998).

### Determination of $pH_i$

The relationship between the medium pH or $pH_e$ and $pH_i$ in HL-60 cells was investigated. The $pH_i$ was determined using BCECF-AM as $pH_i$ indicator (Kim et al, 1991; Park et al, 1996). Cells were suspended in pH 7.5 Tris–MOPS buffered RPMI-1640 medium at a concentration of $10^7$ cells $ml^{-1}$ and incubated with 5 μg $ml^{-1}$ of BCECF-AM at 37°C for 30 min. The labelled cells were washed and transferred into microcentrifuge tubes containing different pH media and incubated for 60–120 min at 37°C. After centrifugation, the cells were resuspended in cuvettes containing $Na^+$- and $HCO_3^-$-free choline chloride buffer at the same pH at which the cells were incubated. The fluorescence intensity of the cells was determined at excitation wavelengths of 441 nm and 505 nm and an emission wavelength of 530 nm. The $pH_i$ was obtained from the ratio of fluorescence intensities at 505 and 441 μm excitation and calibration curves.

### RESULTS

### DNA fragmentation

The apoptotic DNA fragmentation in HL-60 cells incubated for 6 h in different pH media are shown in Figure 1. The DNA was fragmented slightly in pH 7.0 medium but markedly in pH 6.4–6.6 media. The magnitude of DNA fragmentation then declined as the medium pH was further decreased to 6.2 and no DNA fragmentation occurred in media with pH 6.0 or lower. Figure 2 shows the effect of ICE inhibitor, Ac-YVAD-H, and CPP32 inhibitor, Ac-DEVD-H, on the DNA fragmentation. HL-60 cells were pretreated with the inhibitors for 1 h and then incubated for 4 h in pH 6.4 medium. Whereas considerable DNA fragmentation occurred in the control cells, DNA did not fragment when cells were pretreated with the caspase inhibitors before incubating the cells in pH 6.4 medium.

### Cleavage of PARP by acidic stress

Figure 3 illustrates that incubation of HL-60 cells for 6 h in acidic media resulted in cleavage of PARP to a 85 KDa fragment. PARP cleavage was significant in pH 6.6 medium and it further increased in pH 6.4 medium and then diminished sharply in pH 6.2 medium. Densitometric analysis showed that the ratios of the amount of 116 KDa PARP and 85 KDa fragment in pH 6.6, 6.4 and 6.2 media were 1:0.4, 1:1.3 and 1:0.2 respectively. Figure 4 demonstrates that PARP cleavage which occurred after 4 h incubation in pH 6.4 medium was almost completely blocked when the cells were pretreated with either an inhibitor of ICE (Ac-YVAD-H) or an inhibitor of CPP32 (Ac-DEVD-H) for 1 h before exposing the cells to the acidic medium.

© 1999 Cancer Research Campaign 

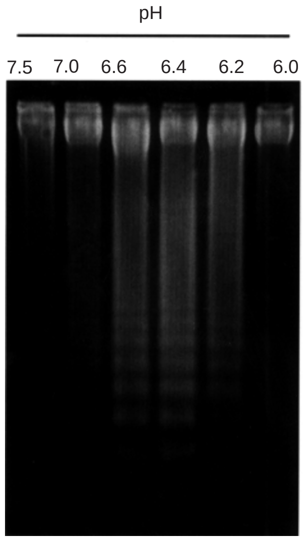

**Figure 1** DNA fragmentation in HL-60 cells incubated in different pH media. After incubating the cells for 6 h at 37°C, DNA was extracted and subjected to agarose gel electrophoresis. The DNA fragmentation was maximal in pH 6.4 medium, in which the $pH_i$ was 6.85 (see Table 1)

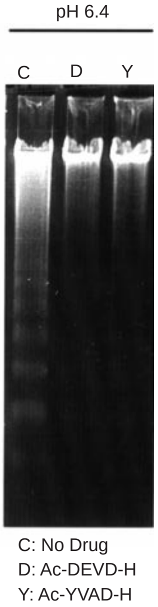

**Figure 2** Effect of caspase inhibitors on acidic stress-induced DNA fragmentation in HL-60 cells. Cells were incubated with 100 μM of Ac-DEVD-H or 100 μM of Ac-YVAD-H for 1 h in pH 7.5 medium and washed. The treated cells were then incubated in pH 6.4 medium for 4 h, DNA was extracted and subjected to agarose gel electrophoresis. The caspase inhibitors blocked the acidic stress-induced DNA fragmentation, which is downstream of PARP cleavage caused by ICE and CPP32

### Flow cytometric analysis of apoptosis

An example of flow cytometric histogram for DNA content in the HL-60 cells incubated in different pH media for 6 h is shown in Figure 5. Table 1 shows the percent of cells in different cell cycle phases and that in apoptosis calculated from the flow cytometric histograms. As can be seen in Figure 5, the apoptotic cell population, i.e. cells with sub-G1 DNA content, increased considerably in pH 6.6 medium and it further increased in pH 6.4 medium. The apoptotic cell population then declined to almost the control level as the medium pH was further lowered to 6.0. The average of 5–10

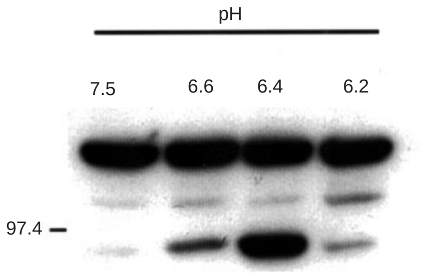

**Figure 3** PARP cleavage in HL-60 cells incubated in different pH media. The cells were incubated in the media for 4 h at 37°C and PARP cleavage was analysed by Western blotting. PARP cleavage occurred in the cells incubated in pH 6.2–6.6 media with a maximal cleavage occurring in pH 6.4 medium. The $pH_i$ of the cells in pH 6.4 medium was 6.86 (see Table 1)

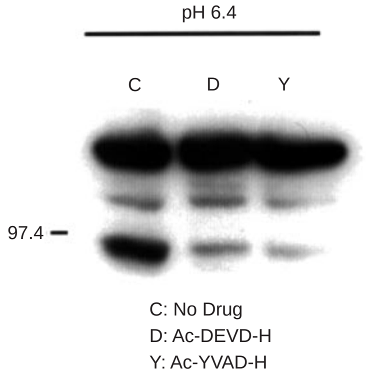

**Figure 4** Effect of caspase inhibitors on the acidic stress-induced PARP cleavage. The cells were incubated with 100 μM of Ac-DEVD-H or 100 μM of Ac-YVAD-H for 1 h and washed. The treated cells were then incubated in pH 6.4 medium for 4 h and PARP cleavage was analysed by Western blotting. The acidic stress-induced PARP cleavage was almost completely blocked by the inhibitors experiments shown in Table 1 demonstrates that 3.4% of cells were in apoptosis in pH 7.5 medium, while 13.2% and 41.9% of cells were in apoptosis after 6 h incubation in pH 6.6 medium and in pH 6.4 medium respectively. These results are consistent with the results of DNA fragmentation study shown in Figure 1. Note that the increase in apoptotic cell population in acidic medium occurred with a concomitant decline in G1 cell fraction suggesting that G1 cells underwent apoptosis. The decline in S cell fraction and G2/M cell fraction after incubation in acidic media may be attributed in part to a decrease in the progression of G1 cells into S phase and G2/M phase.

### Bcl-2 and Bax protein levels

The levels of Bcl-2 and Bax proteins in HL-60 cells after incubation in different pH media are shown in Figure 6. The Bcl-2 protein level was not altered in acidic medium. On the other hand, the Bax protein level was up-regulated within 2 h of incubation in pH 6.4 and pH 6.6 medium and it remained elevated until 6 h of incubation, the extent of our investigation.

### Intracellular pH at different pH media

Table 2 shows the relationship between $pH_i$ and $pH_e$ in HL-60 cells. The $pH_i$ of cells incubated in pH 7.5 medium was lower than

 *© 1999 Cancer Research Campaign*

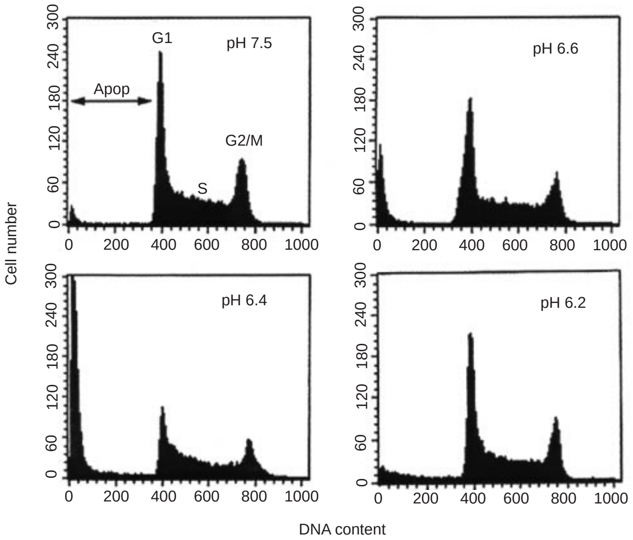

**Figure 5** DNA histogram (**A**) and % of cell distribution in apoptosis and cell cycle phases (**B**) of HL-60 cells were analysed by flow cytometry method. The cells were incubated in media of different pHs for 6 h, fixed with 70% ethanol, stained with propidium iodide, and their DNA content was analysed with flow cytometry. The population of apoptotic cell (cells with sub-G1 DNA content) markedly increased upon incubation in pH 6.4

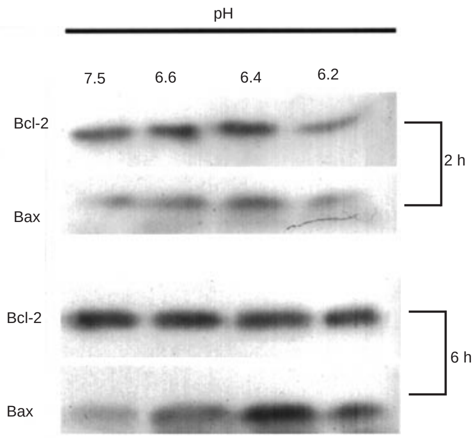

**Figure 6** Expression of Bcl-2 and Bax proteins in an acidic environment. Cells were incubated in different pH media for 2 or 6 h at 37°C. Cellular proteins were subjected to Western blotting analysis. Whereas Bax protein level increased Bcl-2 protein level remained unchanged upon incubation of cells in pH 6.4–6.6 media

**Table 1** The percentage of cells in different cell cycle phases and apoptosis

| | Cell distribution (%) | | | |
|---|---|---|---|---|
| **pH** | **Apoptosis** | **G1** | **S** | **G2/M** |
| 7.5 | 3.44 ± 0.23 | 38.05 ± 1.17 | 33.41 ± 1.03 | 23.50 ± 0.50 |
| 6.6 | 12.23 ± 0.62 | 36.56 ± 0.32 | 30.16 ± 0.38 | 19.50 ± 0.54 |
| 6.4 | 41.92 ± 2.50 | 16.66 ± 1.17 | 26.58 ± 0.98 | 14.43 ± 0.32 |
| 6.2 | 8.85 ± 0.41 | 35.23 ± 0.22 | 34.79 ± 0.34 | 20.36 ± 0.24 |

Average of 5–10 experiments with 1 s.e.m. are shown.

**Table 2** Relationship between pHe and pHi

| pHe | pHi |
|---|---|
| 7.5 | 7.30 ± 0.02 |
| 7.2 | 7.33 ± 0.02 |
| 7.0 | 7.25 ± 0.03 |
| 6.6 | 7.05 ± 0.04 |
| 6.4 | 6.86 ± 0.02 |
| 6.2 | 6.61 ± 0.04 |

The cells were labelled with BCECF-AM, incubated for 120 min in media at different pHs ($pH_e$) and $pH_i$ was determined from the fluorescence intensity.

the medium pH, i.e. $pH_e$. On the other hand, the $pH_i$ was higher than the $pH_e$ when the $pH_e$ was lower than 7.2. For example, the $pH_i$ of HL-60 cells in pH 6.4 medium and pH 6.6 medium, in which apoptosis occurred, was 6.86 and 7.05 respectively.

### DISCUSSION

An incubation of HL-60 cells in pH 6.2–6.6 media for 4–6 h caused apoptosis as judged from PARP cleavage, DNA fragmentation and an increase in cell population with sub-G1 DNA content. The incubation of cells in acidic media for as short as 2 h increased Bax protein level without changing Bcl-2 protein level. It appeared that acidic stress first up-regulates Bax, which in turn increases caspases activity resulting in PARP cleavage and DNA fragmentation.

The acidic stress-induced DNA fragmentation (Figure 1), PARP cleavage (Figure 3) and elevation of Bax protein level (Figure 6) were most pronounced in pH 6.4 medium. Note that the $pH_i$ of HL-60 cells in pH 6.4 medium was 6.86, as shown in Table 2. It should be noted that apoptosis occurs even in pH 6.8–7.0 media when the incubation period is prolonged. We previously reported that apoptosis occurred in HL-60 cells even in pH 7.5 medium when their $pH_i$ was lowered to 6.7–6.9 by inhibiting the $pH_i$ regulatory mechanisms using a combination of inhibitors of $Na^+/H^+$ antiport and $HCO_3^-/Cl^-$ exchange and also $K^+$ ionophore nigericin (Park et al, 1996). Furlong et al (1997) recently reported that IL-3-dependent BAF3 cells underwent apoptosis when IL-3 was removed from the culture medium and also when the cells were treated with topoisomerase inhibitor etoposide or when the $pH_i$ was lowered with nigericin, $K^+$ ionophore. These investigators reported that $pH_i$ decreased during apoptosis and that the general inhibitor of ICE-like proteases ZVAD inhibited the PARP cleavage, but the protease inhibitor did not inhibit the intracellular acidification. It was therefore concluded that the apoptosis signals first caused intracellular acidification, which then directly or indirectly stimulated ICE-like proteases. Contrary to this observation, Wolf et al (1997) recently reported that the caspase inhibitor ZVAD inhibited not only PARP cleavage and DNA fragmentation but also the intracellular acidification during etoposide-induced apoptosis in human ML-1 cells implying that activation of ICE-like proteases is not caused by intracellular acidification. In our present study, apoptosis was induced in HL-60 cells by incubating the cells in acidic medium with no additional treatment or drugs. The observations that intracellular acidification alone caused PARP cleavage and DNA fragmentation, and these events could be effectively blocked with caspase inhibitors (Figures 2 and 4) unequivocally demonstrated that the acidic intracellular environment was responsible for the activation of caspases.

*© 1999 Cancer Research Campaign*

To elucidate whether the caspase activity is stimulated directly or indirectly by intracellular acidification, we determined the levels of Bcl-2 protein and Bax protein. It has been known that Bcl-2 suppresses caspase activity whereas its family member Bax stimulates caspase activity and induces apoptosis and that the expression of these proteins is probably regulated by p53 (Craig, 1995). Although HL-60 cells lack p53 genes (Wolf and Rotter, 1985), an incubation of HL-60 cells in acidic medium increased the level of Bax protein without changing the level of Bcl-2 protein level (Figure 6). Importantly, Bax protein level was increased within 2 h of incubation, whereas it took about 4 h for the appearance of PARP cleavage and DNA fragmentation upon incubation in acidic medium. These results suggested that acidic stress first increases the level of Bax protein thereby stimulating the caspases in the HL-60 cells. CPP32 has been demonstrated to be the major caspase for PARP cleavage and ICE is relatively ineffective in directly causing PARP cleavage. It is unclear why the inhibitors of both ICE and CPP32 could effectively block the cleavage of PARP in the present study (Figure 4). In cells, CPP32 and other ICE-like caspase family members exist as pro-enzymes and activated by interacting with other caspases (Lazebnik et al, 1994; Darmon et al, 1995; Ramage et al, 1995; Duan et al, 1996; Kumar and Lavin, 1996; Takahashi and Earnshaw, 1996). We propose that in acidic environment ICE is activated first, which then activates pro-CPP32 to CPP32 so that either inhibitor of ICE and CPP32 could block PARP cleavage. The optimal pH of CPP32 to cleave PARP has been reported to be 6.5–7.0 (Nicholson et al, 1995; DW Nicholson, personal communication). In this connection, it is important to note that PARP cleavage was most pronounced in pH 6.4–6.6 media, which lowers the $pH_i$ of HL-60 cells to 6.9–7.1 (Table 1). We may then infer that acidic environment not only activates the caspases but also increases the proteolytic process mediated by the caspases. Although PARP cleavage is a common feature in apoptosis, the importance of PARP cleavage in apoptosis is not yet clear because, in addition to PARP, other proteins such as histone, lamins, topoisomerases and DNA-dependent protein kinase (DNA-PK) are also degraded by caspases during apoptosis (Kaufmann et al, 1993; Casciola-Rosen et al, 1995; Ramage et al, 1995; Song et al 1996). Indications are that degradation of these proteins either triggers apoptosis or renders DNA sensitive to endonucleases and that the relative importance of the cleavage of different proteins is cell type-dependent. In this regard, PARP has been demonstrated to suppress the activity of $Ca^{2+}$/$Mg^{2+}$-dependent DNAase I (Yoshihara et al, 1975; Negri et al, 1993; Whitacre et al, 1995; Kumar and Lavin, 1996) suggesting that PARP cleavage may lead to activation of DNAase I. The optimal pH for DNAase I has been known to be in the 6.8–7.0 range suggesting that an acidic environment may enhance the activity of this endonuclease to fragment DNA. It has also been recently reported that CPP32 cleaves ICAD, a complex of caspase-activated deoxyribonuclease (CAD) and its inhibitor (I) in the cytosol, allowing the $Mg^{2+}$-dependent CAD to enter the nucleus and degrade chromosomal DNA (Enari et al, 1998; Sakahira et al, 1998). Another $Mg^{2+}$-dependent endonuclease (AN34) has recently been purified from etoposide-treated HL-60 cells (Yoshida et al, 1998). Caspase inhibitors could inhibit the activity of this DNAase. When these reports are taken together, one may conclude that not a single nuclease but multiple endonucleases may be involved in DNA degradation in apoptosis and that activation of caspases is a critical upstream event of the activation of these endonucleases. In a study by Zanke et al (1998), tumour cells were treated with inhibitors of $pH_i$ regulatory mechanisms in pH 6.0–6.5 media, and the relationship between Bax expression, PARP cleavage and clonogenicity of the cells was investigated. Wherease Bax was up-regulated, PARP cleavage was not observed and the clonogenicity of the cells was not affected by the inhibitors of caspases in the cells received the acid shock. This results are essentially in good agreement with our previous observation (Park et al, 1996) that treatment of HL-60 cells with inhibitors of $pH_i$ regulation caused less apoptotic DNA fragmentation in pH 6.6 medium than in pH 7.5 medium. There is an optimal pH range for the PARP cleavage and DNA degradation and it appears that an extremely acidic environment may kill the cells by mechanisms without involvement of PARP cleavage and apoptotic DNA fragmentation. In the aforementioned study by Zanke et al (1988), it is highly likely that the treatment of the cells with inhibitors of $pH_i$ regulation in media with pH < 6.5 decreases the $pH_i$ significantly lower than 6.5. Under such an extremely acidic intracellular environment, cleavage of PARP with caspases, whose optimal pH is known to be above 6.5 (Nicholson et al, 1995), may not occur. In our present study, we incubated the cells in pH 6.6 medium without any additional stress and the cells underwent apoptosis through PARP cleavage. Experiment is in progress in our laboratory to reveal whether the inhibition of PARP cleavage with caspase inhibitors prevents the acidic stress-induced clonogenic cell death.

There have been considerable discussions regarding in which cell cycle stage apoptosis occurs after receiving various signals for apoptosis (Dewey et al, 1995). The results shown in Figure 5 indicate that mainly the G1 cell fraction decreases while the apoptotic cell population increases upon incubation of the cells in acidic medium indicating that G1 cells undergo apoptosis. In this regard, we previously observed that heat-induced apoptosis in HL-60 cells occurred in G1 phase (Takasu et al, 1998). The effects of acidic intra-tumour environment on the viability and proliferation kinetics of tumour cells and thus on the tumour growth rate remain to be investigated.

### ACKNOWLEDGEMENTS

The authors are thankful to Dr Seymour H Levitt for his continuous encouragement and Ms Peggy Evans for her editorial assistance. This work was supported by grant CA13353 from NCI, an American Cancer Society Institutional grant/IRG-01 and Grant from Inha University Research Foundation (1996).

### REFERENCES

Aisenberg AC (1961) The anaerobic and aerobic glycolysis of normal and tumor tissues. In: *The Glycolysis and Respiration of Tumors*, pp. 1–53. Academic Press, New York.

Casciola-Rosen LA, Anhalt GJ and Rosen A (1995) DNA-dependent protein kinase is one of a subset of autoantigens specifically cleared early during apoptosis. *J Exp Med* **182**: 1625–1634

Chu G and Dewey WC (1988) The role of low intracellular or extracellular pH in sensitization to hyperthermia. *Radiat Res* **114**: 154–167

Craig RW (1995) The bcl-2 gene family. *Semin Cancer Biol* **6:** 35–43

Darmon AJ, Nicholson DW and Bleackley RC (1995) Activation of the apoptotic protease CPP32 by cytotoxic T-cell-derived granzyme B. *Natural* **377**: 466–448

Darzynkiewicz A, Bruno S, Bino GD, Gorczyca W, Hotz MA, Lassota P and Traganos F (1992). Feature of apoptotic cells measured by flow cytometry. *Cytometry* **13**: 795–808

 *© 1999 Cancer Research Campaign*

Dewey WC, Ling CC and Meyn RE (1995) Radiation-induced apoptosis: relevance to radiotherapy. *Int J Radiat Oncol Biol Phys* **33:** 781–796

Duan H, Chinnaiyan AM, Hudson PL, Wing JP, He WW and Dixit VM (1996) ICE-AP3, a novel mammalian homologue of the *Caenorhabditis elegans* cells death protein Ced 3, is activated during Fas- and tumor necrosis factor-induced apoptosis. *J Biol Chem* **271**: 1621–1625

Enari M, Sakahira H, Yokoyama H, Iwamatsa A and Nagata S (1998) A caspase-activated DNase that degrades DNA during apoptosis, and its inhibitor/CAD. *Nature* **391**: 43–50

Furlong IJ, Ascaso R, Rivas AL and Collins MKL (1997) Intracellular acidification induces apoptosis by stimulating ICE-like protease activity. *J Cell Science* **110**: 653–661

Griffiths JJ (1991) Are cancer cells acidic? *Br J Cancer* **64**: 425–427

Gullino PM (1975) Extracellular compartments of solid tumors. In *Biology of Tumors: Cellular Biology and Growth*, Backer JJ (ed) Vol. 3: Cancer, pp. 327–354. Plenum Press: New York.

Haveman J (1979) The pH of the cytoplasm as an important factor in the survival of vitro cultured malignant cells after hyperthermia. Effects of carbonylcyanide-3-chlorophylhydrazone. *Eur J Cancer* **15**: 1281–1288

Jahde E, Glusenkamp KH, Klunder I, Hulser DF, Tietze LF and Rajewsky MF (1989) Hydrogen ion-mediated enhancement of cytotoxicity of bis-chlorethylating drug in rat mammary carcionoma cells in vitro. *Cancer Res* **49**: 2965–2972

Kaufmann SH, Desnoyers S, Ottaviano Y, Davidson NE and Poirier GG (1993) Specific proteolytic cleavage of poly(ADP)-ribose) polymerase: an early marker of chemotherapy-induced apoptosis. *Cancer Res* **53**: 3976–3985

Kim GE, Lyons JC, Levitt SH and Song CW (1991) Effects of amiloride on intracellular pH and thermosensitivity. *Int J Radiat Oncol Biol Phys* **20**: 541–549

Kumar S and Lavin MF (1996) The ICE family of cysteine proteases as effectors of cell death. *Cell Death Differ* **3**: 255–267

Lazebnik YA, Kaufman SH, Desnoyers S, Poirier GG and Earnshaw WC (1994) Cleavage of poly (ADP-ribose) polymerase by a proteinase with properties like ICE. *Nature* **371**: 346–347

Lee HS, Park HJ, Lyons JC, Griffin RJ, Auger EA and Song CW (1997) Radiation-induced apoptosis in different pH environments in vitro. *Int J Radiat Oncol Biol Phys* **38**: 1079–1087

Liu JCK and Fox MH (1995) Modification of intracellular pH and thermotolerance development by amiloride. *Int J Hyperthermia* **11**: 511–523

Liu F-F, Sherar MD and Hill RP (1996) The relationship between intracellular pH and heat sensitivity in a thermoresistant cell line. *Radiat Res* **145**: 144–149

Negri C, Bernardi R, Braghetti A, Ricotti GC and Scovassi AI (1993) The effect of the chemotherapeutic drug VP-16 on poly(ADP-ribosylation) in apoptotic HeLa cells. *Carcinogenesis* **14**: 2559–2564

Nicholson DW, Ali A, Thornberry NA, Vaillancourt JP, Ding CK, Gallant M, Gareau Y, Griffin PR, Labelle M, Lazebnik YA, et al. (1995). Identification and inhibition of the ICE/CED-3 protease necessary for mammalian apoptosis. *Nature* **376**: 37–43

Park HJ, Makepeace CM, Lyons JC and Song CW (1996) Effect of intracellular acidity and ionomycin on apoptosis in HL-60 cells. *Eur J Cancer* **32A**: 540–546

Ramage P, Cheneval D, Chvei M, Graff P, Hemming R, Heng R, Kocher HP, Mackenzie A, Memmert K, Revesz L et al. (1995) Expression, refolding and autocatalytic proteolytic processing of the interleukin-1β-converting enzyme precursor. *J Biol Chem* **270**: 9378–9383

Sakahira H, Enari M and Nagata S (1998) Cleavage of CAD inhibitor in CAD activation and DNA degradation during apoptosis. *Nature* **391**: 96–99

Schlegel J, Peters I, Orrenius S, Miller DK, Thornberry NA, Yamin T-T and Nicholson DW (1996) CPP32/Apopain is a key Interleukin 1β converting enzyme-like protease involved in Fas-mediated apoptosis. *J Biol Chem* **271**: 1841–1844

Song CW, Lyons JC, Griffin RJ, Makepeace CM and Cragoe EJ Jr (1993*a*) Increase in thermosensitivity of tumor cells by lowering intracellular pH. *Cancer Res* **53**: 1599–1601

Song CW, Lyons JC and Luo Y (1993b) Intra- and extracellular pH in solid tumors: Influence on therapeutic response. In: *Drug Resistance in Oncology*, B Teicher (ed), pp. 25–51. Marcel Dekker: New York

Song CW, Kim GE, Lyons JC, Makepeace CM, Griffin RJ, Rao GH and Cragoe Jr EJ (1994) Thermosensitization by increasing intracellular acidity with amiloride and its analogs. *Int J Radiat Oncol Biol Phys* **30**: 1161–1169

Song Q, Lees-Miller SP, Kumar S, Zhang ZN, Chan DW, Smith GC, Jackson SP, Alnemri ES, Litwack G, Khanna KK et al. (1996) DNA-dependent protein kinase catalytic subunit: a target for an ICE-like protease in apoptosis. *EMBO. J.* **15**: 3238–3246

Takahshi A and Earnshaw WC (1996) ICE-related proteases in apoptosis. *Curr Opin Genet Dev* **6**: 50–55

Takasu T, Lyons JC, Park HJ and Song CW (1998) Apoptosis and perturbation of cell cycle progression in an acidic environment after hyperthermia. *Cancer Res* **58**: 2504–2508

Tannock IF and Rotin D (1989) Acid pH in tumors and its potential for therapeutic exploitation. *Cancer Res* **49**: 4373–4384

Whitacre CM, Hashimoto H, Tsai ML, Chatterjee S, Berger SJ and Berger NA (1995) Involvement of NAD-poly(ADP-ribose) metabolism in p53 regulation and its consequence. *Cancer Res* **55**: 3697–3701

Wike-Hooley JL, Haveman J and Reinhold HS (1984) The relevance of tumour pH to the treatment of malignant disease. *Radiother Oncol* **2**: 344–366

Wolf CM, Reynolds JE, Morana SJ and Eastman A (1997) The temporal relationship between protein phosphatase, ICE/CED-3 proteases, intracellular acidification, and DNA fragmentation in apoptosis. *Exp Cell Res* **230**: 22–27

Wolf D and Rotter V (1985) Major deletions in the gene encoding the p53 tumor antigen causes lack of p53 expression in HL-60 cells. *Proc Natl Acad Sci USA* **82**: 790–794

Yoshida A, Pourquier P and Pommier Y (1998) Purification and characterization of a $Mg^{2+}$-dependent endonuclease (AN34) from etoposide-treated human leukemia HL-60 cells undergoing apoptosis. *Cancer Res* **58**: 2576–2582

Yoshihara K, Tanigawa YK, Burzio L and Koide SS (1975) Evidence of adenosine diphosphate ribosylation of $Ca^{2+}$, $Mg^{2+}$-dependent endonuclease. *Proc Natl Acad Sci USA* **72**: 289–293

Zanke BW, Lee C, Arab S and Tannock IF (1998) Death of tumor cells after intracellular acidification is dependent on stress-activated protein kinases (SAPK/JNK) pathway activation and cannot be inhibited by Bcl-2 expression or interleukin 1 β-converting enzyme inhibition. *Cancer Res* **58**: 2801–2808

*© 1999 Cancer Research Campaign*
